# Pathogenesis and pathophysiology of idiopathic normal pressure hydrocephalus

**DOI:** 10.1111/cns.13526

**Published:** 2020-11-26

**Authors:** Zhangyang Wang, Yiying Zhang, Fan Hu, Jing Ding, Xin Wang

**Affiliations:** ^1^ Department of Neurology Zhongshan Hospital, Fudan University Shanghai China; ^2^ Department of Neurosugery Zhongshan Hospital, Shanghai Medical College, Fudan University Shanghai China; ^3^ Department of The State Key Laboratory of Medical Neurobiology, The Institutes of Brain Science and the Collaborative Innovation Center for Brain Science Fudan University Shanghai China

**Keywords:** cerebrospinal fluid dynamics, idiopathic normal pressure hydrocephalus, pathogenesis, pathophysiology

## Abstract

Idiopathic normal pressure hydrocephalus (iNPH), the most common type of adult‐onset hydrocephalus, is a potentially reversible neuropsychiatric entity characterized by dilated ventricles, cognitive deficit, gait apraxia, and urinary incontinence. Despite its relatively typical imaging features and clinical symptoms, the pathogenesis and pathophysiology of iNPH remain unclear. In this review, we summarize current pathogenetic conceptions of iNPH and its pathophysiological features that lead to neurological deficits. The common consensus is that ventriculomegaly resulting from cerebrospinal fluid (CSF) dynamics could initiate a vicious cycle of neurological damages in iNPH. Pathophysiological factors including hypoperfusion, glymphatic impairment, disturbance of metabolism, astrogliosis, neuroinflammation, and blood‐brain barrier disruption jointly cause white matter and gray matter lesions, and eventually lead to various iNPH symptoms. Also, we review the current treatment options and discuss the prospective treatment strategies for iNPH. CSF diversion with ventriculoperitoneal or lumboperitonealshunts remains as the standard therapy, while its complications prompt attempts to refine shunt insertion and develop new therapeutic procedures. Recent progress on advanced biomaterials and improved understanding of pathogenesis offers new avenues to treat iNPH.

## Graphical abstract

In this review, we summarized the pathogenesis of idiopathic  normal  pressure hydrocephalus (iNPH) and its pathophysiological features that lead to neurological deficits. We also reviewed the current treatment strategies for iNPH and discussed prospective future therapies.

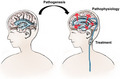

## 1 INTRODUCTION

First defined in 1965, idiopathic normal pressure hydrocephalus (iNPH)is a surgically reversible neurological disorder in adults. It is characterized by dementia, gait disturbance, and urinary incontinence (known as Hakim's triad).^1^, ^2^ INPH is not a rare clinical entity. The prevalence of iNPH has been estimated to be 10 per 100 000 to 22 per 100 000 overall, with 1.30% in those aged ≥65 years and 5.9% in those aged ≥80 years.^3^, ^4^ One of the core features of iNPH is that the cerebrospinal fluid (CSF) pressure of an iNPH patient is within normal ranges.^2^ Typical brain imaging of iNPH displays ventriculomegaly, periventricular hyperintensities, wide Sylvian fissures, narrowed subarachnoid space, and cortical sulci at the high convexity.^5^ In contrast to the secondary normal pressure hydrocephalus with known etiology, the exact cause of iNPH is unknown.^6^ Its specific pathogenesis also remains elusive, although several mechanisms have been proposed to contribute to the development of iNPH.^7^ Treatment of ventricular shunting, mostly the ventriculoperitoneal shunting, was proved to be successful in alleviating symptoms in approximately 60%‐80% patients.^8^, ^9^ This review updates on the pathogenesis and pathophysiology of iNPH, including abnormal CSF dynamics and neurological deficits, and recent advances in the treatment strategies for iNPH.

## 2 METHODS

We searched Medline/PubMed database for the relevant literature using the using the Medical Subject Headings (MeSH) terms “Hydrocephalus, Normal Pressure,” and following keywords “pathogenesis,” “pathophysiology”. The titles and abstracts were reviewed. And 78 articles pertaining to the INPH were included. Non‐English language articles; gray literature publications; editorials and commentaries were excluded. Additional references were obtained from bibliography of selected articles.

## 3 ABNORMAL CSF

In the physiological condition, CSF flow has both bulk and pulsatile components. Driven by the continuous production of CSF, the CSF bulk flow moves from lateral ventricles to the third ventricle through the interventricular foramen. It continues to flow across the aqueduct of Sylvius down to the fourth ventricle. Further, through the foramen of Magendie and foramen of Luschka, CSF flows to the subarachnoid space where it is reabsorbed into blood circulation.^10^ On the contrary, CSF pulsatile flow is driven by arterial pulsatility and intracranial blood inflow. It is most prominent in the aqueduct of Sylvius, representing a rapid CSF oscillation.^11^ Closely regulated by cardiovascular pulsation, CSF pulsatile flow moves through aqueduct from the third ventricle to the fourth ventricle during systole and flows backward during the diastole.^12^

Abnormal CSF dynamics has been a major research focus in the etiology of iNPH.^11^ It was believed to be the initiating factor that contributes to the subsequent ventriculomegaly and neurological deficits in iNPH.^6^, ^13^ Mechanisms concerning the disturbance of CSF pulsatility and normal CSF drainage have been proposed.

### 3.1 Increased CSF pulsatility

It has long been observed on MRI images of iNPH patients that the aqueduct flow voids might be an indicator of hyperdynamic CSF flow through the aqueduct.^5^ Later application of phase‐contrast (PC)‐MRI enables the observation and quantitation of CSF flow within a cardiac cycle without the injection of tracers.^14^ Using PC‐MRI, the measurement of aqueduct stroke volume (ASV), which is defined as the average of flow volume through the aqueduct during diastole and systole, may represent the CSF pulsatility.^15^, ^16^ There are various PC‐MRI studies showing increased ASV in iNPH patients compared to healthy controls.^17^, ^18^, ^19^, ^20^ Luetmer et al^21^ demonstrated that elevation of ASV assisted the diagnosis of iNPH and distinguished iNPH from other types of dementia. In a serial PC‐MRI study, Scollato et al^22^ found a progressive increase of ﻿ASV for 18 to 20 months along with the progression of iNPH symptoms. Also, comparison between shunt responders and shunt non‐responders revealed that patients with higher ASV may benefit more from shunting operation.^22^ Similarly, other flow‐related parameters such as CSF velocity, pressure gradient, and rotation also significantly elevated in iNPH patients, demonstrating a hyperdynamic state of CSF motion.^11^, ^20^, ^23^, ^24^ Increased CSF pulsatility may associate with reduced arterial pulsatility and decreased intracranial compliance.^19^, ^25^, ^26^, ^27^ The aqueduct pulsatility reflects capillary expansion, which is mainly influenced by the pulsatile dampening from the artery according to the Windkessel mechanism.^13^, ^28^ In the aging process, the loss of arterial pulsatility due to atherosclerosis would significantly increase the pulsatility of aqueduct.^25^ In the meantime, intracranial compliance decreases with aging and neurodegeneration, which would restrict the CSF motion in rigid CSF places including subarachnoid space and convexity.^29^, ^30^, ^31^ As a result, the pressure gradient elevated evidently in the aqueduct, turning the CSF flow into a hyperdynamic state.^20^ Intriguingly, the direction of hyperdynamic CSF flow is frequently reversed, which tends to flow into the ventricles.^32^ Yin et al studied the CSF flow separately in the systole and diastole phase and found that the increased pulsatile flow though the aqueduct in two phases was not equally matched. The rise of CSF flow in diastole phase exceeds that of systole phase, causing a reversed aqueductal CSF net flow in the direction of caudal‐cranial.^18^ The retrograde aqueductal flow could generate a sustained pressure gradient, leading to compressive stress and shearing forces to the ependyma. It may further contribute to the dilation of ventricles, which is a core anatomical feature of iNPH.^5^, ^20^ Indeed, the causal relationship between ASV and ventricular volume has been confirmed.^33^

### 3.2 Reduced CSF drainage

To fully understand the role of abnormal CSF dynamics in the pathogenesis of iNPH, the concept of abnormal CSF drainage should not be overlooked. It has long been acknowledged that in the normal condition, the CSF in the subarachnoid space is reabsorbed through the arachnoid granulations and taken up into the superior sagittal sinus due to the pressure gradientforce.^30^, ^34^ Recent reports demonstrated that a considerable proportion of CSF could be drained into cervical lymphatic systems.^30^ The disturbance of normal CSF drainage is observed in patients with iNPH. In these patients, the resistance to CSF outflow (Rout) is pathologically elevated and has been widely used for the diagnosis of iNPH and the selection of candidates for shunting surgery.^35^ Boon et al^36^ showed that over 83% iNPH patients had the Rout over 12 mm Hg/mL/min, while the values of healthy controls never exceed 10 mm Hg/mL/min. A meta‐analysis revealed that a Rout of 12 mm Hg/mL/min is a suitable threshold for predicting shunt responsiveness.^37^

Studies about abnormal CSF drainage in iNPH mostly focus on the venous‐related route. Bateman et al^38^ compared the venous outflow volumes between iNPH patients and age‐matched controls. The results reveal significantly reduced venous outflow in iNPH. The authors further showed that this reduction was due to sinuses stenosis and subsequent elevation in venous pressure in iNPH patients.^39^ It is reported that 3‐4 mm Hg rise of sagittal sinus pressure could cease the CSF absorption via the granulations.^40^ The retrograde jugular venous flow found in iNPH could induce transmission of high venous pressure, possibly leading to lower flow velocity at the superior sagittal sinus.^41^ Furthermore, abnormal activation of transependymal CSF absorption in perivascular white matter (PVWM) may serve as a parallel pathway to compensate the resistance of CSF outflow, which may contribute to periventricular white matter hyperintensities.^6^, ^13^ The CSF outflow channels (eg, subarachnoid space and veins) and conductivity are major contributing factors of the CSF outflow resistance.^42^ As aforementioned, aging has a significant influence on the intracranial compliance.^23^ Intracranial compliance gradually reduces with the progress of aging, resulting in more rigid CSF circulatory channels and the elevation of venous pressure. Therefore, it is conceivable that the Rout increases with aging.^43^ Lack of sufficient CSF absorption caused by outflow resistance could lead to the accumulation of CSF, and further facilitate the ventricular dilatation driven by high CSF pulsatility. Meanwhile, elevated venous pressure also reduces intracranial compliance and has considerable influence on the CSF hyperdynamics.^44^

## 4 HYPOPERFUSION LEADS TO NEUROPHYSIOLOGICAL CHANGES IN iNPH

Ventricular enlargement induced by abnormal CSF dynamics increases mechanical stress on the parenchyma and blood vessels, causing hypoperfusion and consequent hypoxia in iNPH.

Multiple imaging modalities including MRI, computed tomography, single photon emission computed tomography, or positron emission tomography (PET) indicate both global and regional CBF reduction in the PVWM, gray matter, and basal ganglia in iNPH patients.^45^, ^46^, ^47^, ^48^ Such reduced CBF in different regions might account for different symptoms of iNPH. For example, it is reported that CBF reduction in the basal ganglia correlated with the severity of gait abnormality.^49^ Right frontal hypoperfusion closely related with urinary dysfunction.^50^

Hypoperfusion could further leads to a series of pathophysiological changes of brain tissue including alterations of metabolism, gliosis, neuroinflammation, and blood‐brain barrier impairments.

### 4.1 Alterations of metabolism

It is generally acknowledged that hypoperfusion and hypoxia could disturb the normal cellular homeostasis, especially the oxygen‐related energy metabolism.^51^, ^52^ Attention has been payed to the metabolic alterations in iNPH.^53^, ^54^, ^55^ It is found using PET imaging that not only the regional cerebral metabolic rate of oxygen, but also the overall glucose metabolism declined significantly in the basal ganglia.^47^, ^56^ As the basal ganglia plays a key role in voluntary motor control and gait regulation, it is speculated that decreased oxygen metabolism and glucose utilization contribute to the gait disturbance in iNPH.^47^, ^56^ Lactate, the end product of anaerobic metabolism, is a sensitive marker of hypoperfusion and hypoxia.^53^ Pathologically high lactate values could be observed in CSF samples from iNPH patient,^53^ which was in line with a study showing that lactate accumulated within the ventricular system after the reduction of periventricular CBF.^55^ However, magnetic resonance spectroscopy did not show any difference in the level of lactate between iNPH patients and controls.^54^ These conflicting results may be attributed to different detecting methods or heterogenous patient groups.

Some metabolites that have been analyzed in iNPH could serve as valuable biomarkers. In iNPH patients, the value of N‐acetylaspartate (NAA)/creatine was correlated with the MMSE score, and concentrations of NAA and total N‐acetylaspartate (tNA) were significantly declined in thalamus compared to healthy controls.^54^ Metabolic changes could also predict prognosis after shunt surgery in iNPH. For example, increased total choline level and decreased myo‐inositol level in the frontal deep white matter indicate clinical improvement in iNPH patients.^57^

### 4.2 Astrogliosis and neuroinflammation

Astrogliosis is evident in iNPH patients. GFAP (glial fibrillary acidic protein) is commonly used as a marker for reactive astrocytes.^58^ Larger GFAP‐stained areas have been observed in the brain tissue specimens collected from iNPH patients compared to those from healthy controls.^58^, ^59^ GFAP levels also elevated in the CSF samples from iNPH patients.^55^ Astrogliosis may worsen the abnormal CSF dynamics by increasing parenchymal stiffness and decreasing the compliance of the brain.^60^, ^61^

Neuroinflammatory responses, which are characterized by the release of inflammatory mediators, such as cytokines and chemokines,^62^, ^63^ are prominent during iNPH. Altered levels of inflammatory cytokines in the CSF, as summarized in Table 1, have been reported.^64^, ^65^, ^66^, ^67^, ^68^, ^69^, ^70^ Among them, TNF‐α is the most extensively studied.^64^, ^67^, ^70^ One study measured TNF‐α level in the CSF before and after shunt operation in iNPH individuals and detected higher intrathecal TNF‐α level in iNPH patients, which could be reversed completely following the shunt surgery.^70^ Furthermore, TNF‐α level was correlated with clinical symptoms, especially the cognitive function decline.^70^ A positive correlation was also observed between the expression of TNF‐α and sulfatide, a group of glycosphingolipids that are highly expressed in the myelin sheath, in the CSF, suggesting detrimental effect of TNF‐α on white matter integrity.^70^ Several other groups confirmed higher CSF levels of TNF‐α in iNPH patients compared with healthy controls or non‐iNPH disease controls.^64^, ^67^ TGF‐β1 level is also elevated in the CSF of iNPH patients compared to patients with tension‐type headache.^68^ TGF‐β1 is known to be upregulated in endothelial cells and connective tissue cells in response to tissue injury or fibrosis.^71^ TGF‐β‐mediated subarachnoid fibrosis is involved in the hydrocephalus after brain hemorrhage.^72^, ^73^ It is unknown whether this subarachnoid fibrosis also contributes to the development of iNPH. Other inflammation modulators, such as monocyte chemoattractant protein‐1 (MCP‐1) and IL‐6, were also changed in the CSF after iNPH^66^, ^67^, ^68^, ^69^, ^74^, ^75^ (Table 1). The exact roles of each inflammatory factor and their interactions in the pathogenesis and pathophysiology of iNPH await further elucidation.

**Table 1:** Cytokines in the pathophysiology of iNPH

Sample	Study design	Sample types	Cytokines	Key findings	Preoperative and postoperative differences	Authors & Year
16 iNPH, 25 HC	Retrospective	CSF	TNF‐α	Higher CSF levels of TNF‐α in iNPH patients compared to HC.	TNF‐α levels decrease after shunt operation.	Tarkowski E,^70^ 2003
6 iNPH, 11 HC, 7 MCI	Retrospective	CSF	TNF‐α	Higher CSF levels of TNF‐α in iNPH patients compared to HC and MCI.	/	Castañeyra‐Ruiz L,^64^ 2016
8 iNPH, 10 SAH‐induced hydrocephalus, 6 non‐hemorrhagic obstructive hydrocephalus	Retrospective	CSF	TNF‐α	Higher CSF levels of TNF‐α in iNPH patients compared to non‐hemorrhagic obstructive hydrocephalus.	/	Lee JH,^67^ 2012
20 iNPH, 20 non‐iNPH DC	Retrospective	CSF, plasma	IL‐1β	Higher CSF levels of IL‐1β in iNPH patients compared to DC.	/	Sosvorová L,^69^ 2014
20 iNPH, 20 non‐iNPH DC	Retrospective	CSF, plasma	IL‐6	Higher CSF levels of IL‐6 in iNPH patients compared to DC.	/	Sosvorová L,^69^ 2014
5 INPH, 2 non‐iNPH DC	Retrospective	CSF	IL‐6	Higher CSF levels of IL‐6 in iINPH patients compared to DC.	/	Czubowicz,^77^ 2017
5 iNPH, 2 non‐iNPH DC	Retrospective	CSF	IL‐8	Higher CSF levels of IL‐8 in iNPH patients compared to DC.	/	Czubowicz,^77^ 2017
20 iNPH, 20 non‐iNPH DC	Retrospective	CSF, plasma	IL‐10	Higher CSF levels of IL‐10 in iNPH patients compared to DC.	/	Sosvorová L,^81^ 2014
28 iNPH, 20 HC	Retrospective	CSF	MCP‐1	Higher CSF levels of MCP‐1 in iNPH patients compared to HC.	MCP‐1 levels increase after shunt operation.	Jeppsson A,^78^ 2013
8 iNPH, 10 SAH‐induced hydrocephalus, 6 non‐hemorrhagic obstructive hydrocephalus	Retrospective	CSF	TGF‐β1	Higher CSF levels of TGF‐β1 in iNPH patients compared to non‐hemorrhagic obstructive hydrocephalus.	/	Lee JH,^67^ 2012
21iNPH and 14 tension‐type headache	Retrospective	CSF	TGF‐β1	Higher CSF levels of TGF‐β1 in iNPH patients compared to tension‐type headache.	/	Li X,^80^ 2007

Abbreviations: CSF, cerebrospinal fluid; DC, disease controls; HC, healthy controls; IL, interleukin; iNPH, idiopathic normal pressure hydrocephalus; MCI, mild cognitive impairment; MCP‐1, monocyte chemoattractant protein 1; SAH, subarachnoid hemorrhage; TGF, transforming growth factor; TNF, tumor necrosis factor.

### 4.3 Loss of Blood‐brain Barrier integrity

Blood‐brain barrier (BBB) refers to the barrier between the blood and the brain parenchyma, forming mainly by tight junction‐sealed brain endothelial cells.^76^, ^77^, ^78^ These endothelial cells interact with other cell types within the neurovascular unit, including astrocytes and pericytes to maintain the homeostatic state of the brain microenvironment.^79^, ^80^ BBB leakage results in the entry of blood‐borne substances into the brain parenchyma.^81^ BBB disruptions have been shown to play a key role in the neurological dysfunction after CNS disorders.^82^, ^83^, ^84^ Eide et al^81^ measured the fibrinogen extravasation, an indicator of BBB leakage, in cortical brain tissue specimens from 45 iNPH patients and 14 reference subjects. The results show more pronounced fibrinogen extravasation in iNPH specimens, indicating severe breach of BBB integrity in iNPH compared to control subjects. Moreover, it is discovered that increased BBB leakage is associated with the extent of astrogliosis.^81^ Distorted and thickened capillary basement membranes, as well as increased number of degenerating pericytes, are also observed in biopsy samples of iNPH patients.^81^, ^85^ These pathological alterations might all contribute to BBB leakage in iNPH. In contrast, CSF‐based research suggests relative preservation of the BBB integrity in iNPH subjects using the CSF/blood albumin ratio as an index of BBB function.^77^ No marked difference in CSF/blood albumin ratio was found between iNPH patients and healthy controls.^66^ Such discrepancy might be due to the difference in the molecular mass between fibrinogen and albumin or the variance in the sensitivity among different methods for BBB leakage assessments. Further investigations with more sensitive and reliable markers of BBB leakage are warranted.

## 5 GLYMPHATIC IMPAIRMENT INCREASES BRAIN DAMAGE IN iNPH

The recently discovered glymphatic system facilitates the bulk flow of CSF into the brain along the para‐arterial space, the interstitial space and eventually into the para‐venous space. This pathway is mediated by Aquaporin‐4 (AQP4) channels at astrocytic perivascular endfeet.^86^ It promotes the clearance of excess fluid and waste metabolites from CNS.^86^ Normal function of glymphatic system relies on appropriate arterial pulsation, intact AQP4 channels and adequate sleep.^86^

Impaired glymphatic system is observed in iNPH individuals. Using contrast‐enhanced MRI, delayed removal of the intrathecal CSF tracer gadobutrol was observed in iNPH.^87^, ^88^ Since clearance of gadobutrol resembles glymphatic clearance of other metabolites, this result provides compelling evidence for the impaired glymphatic function.^87^, ^88^ The underlying mechanisms are diverse. First of all, excessive CSF might stagnate in the dilated perivascular spaces (PVS), which compresses on the penetrating arteries to reduce their pulsatility.^89^ Secondly, decreased AQP4 density in the astrocytic perivascular endfeet is reported in iNPH patients.^90^ In addition, the progressive AQP4 depolarization with aging further exacerbates the abnormal perivascular fluid flow in aged iNPH patients.^91^, ^92^, ^93^, ^94^ Thirdly, sleep disorders are frequently diagnosed among iNPH patients. A study among 31 iNPH subjects revealed that all iNPH patients suffered from sleep abnormality to different extent.^95^ Over 90.3% of iNPH patients had an obstructive sleep apnea.^95^ Sleep abnormality could promote the overnight accumulation of metabolites in the interstitial space. It is hypothesized that repeated inspiratory movements against tongue‐blocked airway lead to elevated intrathoracic negative pressure and reduced venous return, which ultimately cause intracranial venous hypertension and iNPH.^95^

A direct consequence of the glymphatic system disturbance is the reduced clearance of neurotoxic substances, such as beta amyloid (Aβ) and hyperphosphorylated tau (HP tau).^53^, ^87^ Accumulation of these neurotoxic substances could not only impair physiological functions of neurons, but also trigger astrogliosis and neuroinflammation.^96^ Several studies reported the aggregation of Aβ and HP tau in brain biopsy samples from iNPH patients.^97^ This Alzheimer's disease (AD)‐related pathology is related to cognitive decline in iNPH.^98^ Since human studies also implicated impaired glymphatic function in AD, it is speculated that AD and iNPH share common pathogenesis to some extent.^99^

## 6 WHITE MATTER INJURY IN iNPH

Hypoperfusion, glymphatic impairment, and other aforementioned pathophysiological changes in iNPH culminate in brain lesion and functional deficits. White matter lesions are prominent in iNPH.^6^ Symptoms of iNPH could be attributed, at least partially, to the compression and stretch of white matter. When the motor nerve fibers of corticospinal tract are inflicted, gait disturbance may occur. The stretch of sacral fibers of the corticospinal tract may disrupt bladder contractions, resulting in urinary incontinence, which is the second most common symptom of iNPH.^100^, ^101^

White matter lesions in iNPH could be characterized by hyperintensities on T2‐Fluid‐attenuated inversion recovery (FLAIR) MRI imaging, especially the periventricular white matter hyperintensities (PVH).^99^ DTI‐MRI is another useful technique to assess the white matter lesion. Mean diffusivity (MD) and fractional anisotropy (FA) are two commonly analyzed DTI parameters.^96^ MD increases when more free water diffuses in the extracellular space. FA describes the degree of anisotropy of a diffusion process and reflects the structural integrity of the white matter.^102^ Generally, higher MD and lower FA are observed in most white matter‐enriched regions in iNPH patients, displaying white matter edema and the loss of integrity.^103^ Links between DTI indices and clinical manifestations have been reported. For instance, low FA in the frontal and parietal subcortical white matter is related to cognitive dysfunction. Low FA in the anterior limb of the left internal capsule, corpus callosum and below the left supplementary motor area is associated with gait disturbance in iNPH.^103^ In addition, a strong positive correlation between MD in the corticospinal tract and gait abnormalities has been documented.^104^ DTI may also help differentiating iNPH from other neurodegenerative diseases based on the FA and MD values in different white matter regions (eg,﻿Internal capsule and corticospinal tract).^105^, ^106^

Pathologically, damages on both myelin and myelin‐sheathed axons have been detected in iNPH. Neurofilament light chain (NFL) is a protein that functions to maintain axonal architecture. Myelin basic protein (MBP) is a vital structural protein of myelin.^107^, ^108^ NFL and MBP are commonly used to assess axonal and myelin integrity, respectively. In iNPH, many studies observed increased levels of NFL and MBP in CSF samples and revealed positive correlations between the upregulation of NFL and MBP in the CSF and the severity of PVH on MRI.^66^, ^99^ High level of NFL in the CSF is associated closely with clinical symptoms and can be used as a biomarker to measure the severity of axonal loss in iNPH.^99^ Elevated level of MBP in CSF might be used as a biomarker for myelin damage in iNPH.^66^

## 7 GRAY MATTER INJURY IN iNPH

It is known that the axon of an unhealthy neuron may progressively dies back over weeks, beginning distally and spreading proximally toward the cell body.^109^ Therefore, gray matter lesions are also observed in iNPH patients. Ishii et al^110^ reported remarkably decreases in gray matter density in the insula, caudate and thalamus in iNPH patients, as measured by voxel‐based morphometry analysis. They speculated that such gray matter lesions might due to the transmantle pressure that originated from the ventricular dilation to these regions.

﻿The structural and functional organizations in gray matter have been analyzed in iNPH. In a structural network study, larger global network modularity and network decentralization of frontal, temporal, posterior cingulate and insula cortices were observed in iNPH patients compared to controls.^111^ Functional network studies with resting‐state functional MRI showed that the connectivity of default mode network reduced in iNPH, which was associated with clinical symptoms, particularly, cognitive and urinary dysfunctions.^112^ Transcranial magnetic stimulation is commonly used in the functional network studies. Reduced corticospinal excitability and intracortical inhibitory connectivity in frontal and primary motor cortices are thought to be the culprits of gait disturbances in iNPH.^113^

## 8 CURRENT AND PROSPECTIVE TREATMENT STRATEGIES

Cerebrospinal fluid diversion with ventriculoperitoneal or lumboperitoneal shunts is currently the first‐line and standard therapy for iNPH.^8^, ^114^ Efficacy does not differ significantly between two﻿ types of shunting procedures. Approximately 60%‐80% patients are reported to reduce gait disturbance, one of the most shunt‐responsive symptoms. Ventriculoperitoneal shunts have relatively low shunt failure rate, while lumboperitoneal shunts have the advantage of less invasiveness.^115^

The reversal of abnormal CSF dynamics could be the principal mechanism underlying the therapeutic effect of CSF diversion. Drainage of excessive CSF restores normal CSF pulsatility and directly compensates for the insufficient CSF absorption in iNPH patients.^116^ Therefore, it is not a surprise to find out that different CSF flow parameters, such as ASV, Rout, and CSF waveforms, are used to predict the clinical response to the shunting operation.^15^, ^117^, ^118^ For instance, aqueduct stroke volume greater than or equal to 42 L is applied to identify patients who might benefit from the shunt surgery.^119^ Ventricular decompression after CSF diversion further leads to the restoration of regional and global blood perfusion and the normalization of subsequent pathophysiological changes. Besides, CSF diversion increases the pulsatility of penetrating arteries by decompression, improving the glymphatic function.^89^ When surgical intervention is not indicated for a patient, repeated large volume lumbar puncture is an alternative treatment of CSF drainage.^25^

There are currently no FDA‐approved pharmacological treatments for iNPH. Clinical trials suggest that acetazolamide, a carbonic anhydrase inhibitor, can reduce periventricular white matter hyperintensities and improve symptoms in iNPH.^120^ However, the small sample size, retrospective, and open‐label study design confounded their conclusions. Prospective, double‐blind, and placebo‐controlled trials with larger numbers of subjects are warranted to assess the therapeutic effect and safety of acetazolamide.

Recent years have witnessed a better understanding of iNPH pathogenesis and pathophysiology accompanied by bioengineering advances in medical devices. Novel treatment strategies are on the horizon. Shunt obstruction and infection remain critical concerns in iNPH patients with shunt surgery.^115^ According to a recent meta‐analysis, 9%‐16% patients inserted with an adjustable valve and 26%‐38% patients inserted with a fixed‐pressure valve received an additional surgery.^115^ Therefore, novel shunt catheters made with advanced biomaterials are needed to prevent cell and bacterial adhesion. Concomitantly, development of “smart” shunts with telemetry, incorporated monitors, and auto‐regulated valves has the potential to improve the treatment outcome.^121^ To avoid the permanent implantation of shunts, endoscopic third ventriculostomy (ETV), a procedure formerly demonstrated to be effective in obstructive hydrocephalus, is gaining attention as a surgical treatment of iNPH.^122^ In some studies, the ETV had similar or even superior efficacy and lower complication rates comparing to the shunting procedures.^122^ However, due to the small number of patients and lack of randomized controlled trials, the generalizability of ETV in iNPH is still limited. Future studies are warranted to determine if ETV could be used for iNPH treatment to a broader population.

Heterogeneous clinical outcomes after surgical treatments clearly indicate that it is not enough merely to drain the excessive CSF. This procedure does not address all pathophysiological problems underlying iNPH.^115^, ^123^ Novel pharmacological agents could serve as supplementary or alternative therapies in iNPH. According to the pathogenesis of iNPH, pharmacological interventions could be used to normalize CSF hyperdynamics, such as decreasing CSF production, pulsatility and outflow resistance; restoring blood perfusion, promoting clearance of waste metabolites, alleviating neuroinflammation, and providing neuroprotection. Meanwhile, prospective studies using multimodal techniques are needed to identify objective imaging or CSF biomarkers for the quantitative assessments of drug efficacy. In addition, the development of new animal models that better recapitulate the clinical and radiological features of iNPH will definitely pave the way for preclinical screening of potential drugs.

## 9 CONCLUSION

**Figure 1:**
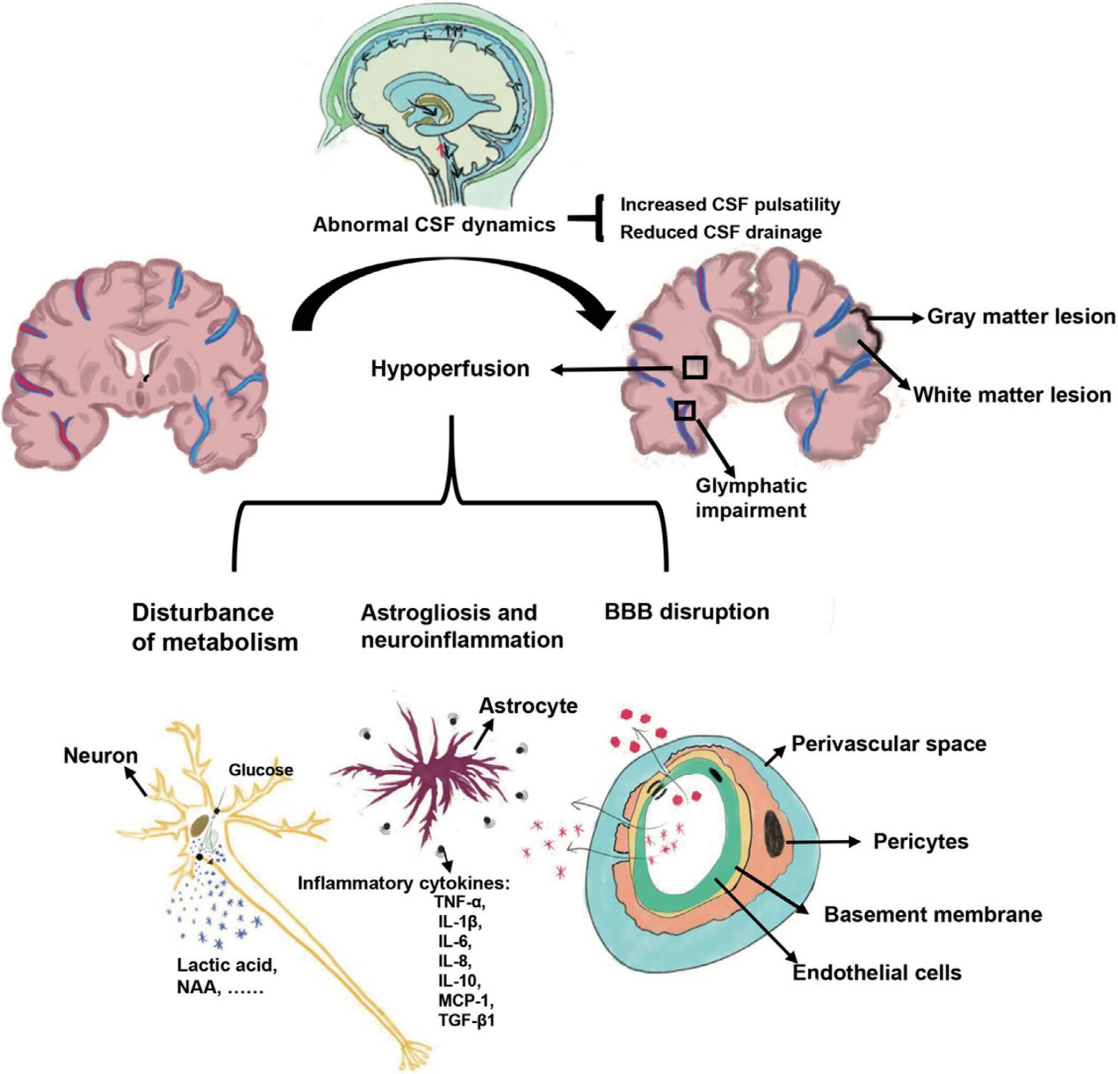
Schematic diagram of pathogenesis and related pathophysiological changes of iNPH. Abnormal CSF dynamics including increased CSF pulsatility and reducedCSF drainage contribute to the chronic development of ventriculomegaly. The subsequent transmantle pressure that originates from the ventricular dilation further leads to regional and global hypoperfusion/hypoxia. This vital pathophysiology initiates a cascade of serial brain damages including disturbance of metabolism, astrogliosis, neuroinflammation, and BBB disruption. Besides, glymphatic impairment, possibly caused by CSF stagnation and other risk factors, contributes to the Alzheimer disease‐like pathology in iNPH. All these factors culminate in white matter and gray matter lesions, which are the basis of clinical manifestations in iNPH. BBB, blood‐brain barrier; CSF, cerebrospinal fluid; IL‐10, interleukin 10; IL‐1β, interleukin 1beta; IL‐6, interleukin 6; IL‐8, interleukin 8; iNPH, Idiopathic normal pressure hydrocephalus; MCP‐1, monocyte chemoattractant protein‐1; NAA: N‐acetylaspartate; TGF‐β1, transforming growth factor‐beta1; TNF‐α, tumor necrosis factor‐alpha

Here, we provide a comprehensive review on the pathogenesis of iNPH and various mechanisms leading to neurological deficits. Instead of being totally idiopathic, etiologic factors and pathophysiological changes might constitute a vicious, deregulating loop that eventually result in clinical symptoms of iNPH (Figure 1). We also updated the current and prospective treatment strategies for iNPH. Despite the heterogeneous responses and possible complications of CSF diversion, it prevents the development of irreversible neurodegeneration and remains to be the first‐line treatment for iNPH. Improved understanding of the iNPH pathogenesis and its relevant neurological deficits, together with the advances in bioengineering, will offer new avenues to refine current treatments and develop novel surgical and pharmacological therapeutic strategies for iNPH.

## CONFLICT OF INTEREST

The authors declare no conflict of interest.
